# A Lip Lump: An Unexpected Histological Diagnosis of a Lip Schwannoma

**DOI:** 10.1155/2017/3107362

**Published:** 2017-02-07

**Authors:** Thomas Haigh, John Raad Glore, David Gouldesbrough, Winson Wong

**Affiliations:** ^1^Bradford Teaching Hospitals NHS Foundation Trust, Bradford BD9 6RJ, UK; ^2^Bradford Teaching Hospitals NHS Foundation Trust, University of Leeds, UK

## Abstract

Schwannomas are benign nerve sheath tumours arising from Schwann cells. They comprise 1% of all benign tumours. In the 2016 World Health Organisation Classification of Central Nervous System, they are classified as a tumour of the cranial and paraspinal nerves, Schwannoma 9560/0. A 23-year-old Caucasian lady presented with a seven-month history of a painless right upper lip lump. Examination revealed a small cystic 0.5 cm diameter lesion within the right upper lip. The clinical impression was that of a mucocele. Excision of the lip lesion was performed under local anaesthetic. Histological examination of the excised lesion demonstrated a circumscribed nodule consisting of spindle cells mixed with vascular spaces containing red blood cells and fibrin. Immunohistochemistry for S100 was strongly positive. The findings were consistent with that of a small benign schwannoma. The current consensus is for surgical excision of a conservative nature with no need for margins. If recurrence does occur one needs to consider whether complete enucleation was achieved or whether malignant transformation has occurred.

## 1. Introduction

Lip lumps can present to both the general practitioner in the community and the specialist in a general ENT clinic following referral. In the case presented the unexpected, rare diagnosis only became clear following excision and histological examination.

## 2. Case Report

A 23-year-old Caucasian lady presented with a seven-month history of a painless right upper lip lump that she caught whilst eating. She possessed no red flag symptoms and no significant medical history apart from being a smoker with poor dentition.

Examination revealed a small cystic 0.5 cm diameter lesion within the right upper lip. Excision of the lip lesion was performed under local anaesthetic. A piece of tissue measuring 7 × 3 × 2 mm was sent for histological staining and examination.

## 3. Diagnosis

### 3.1. Schwannoma

In the 2016 World Health Organisation Classification of Central Nervous System, they are classified as tumour of the cranial and paraspinal nerves, Schwannoma 9560/0 [1].

**Figure 1:**
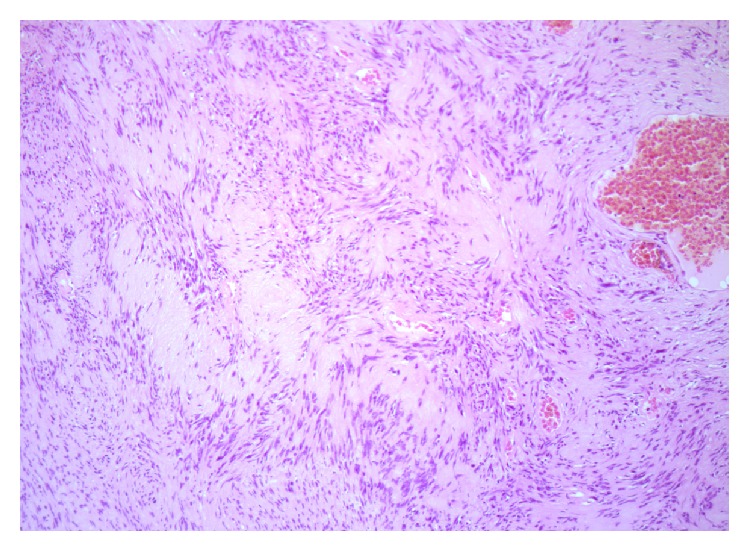
*High-power slide of schwannoma*: H + E ×400. The tumour comprises cytologically bland spindle cells with no mitotic activity or necrosis. Focal palisading of the nuclei is seen as a characteristic of a schwannoma. This has led to the production of the so-called Verocay bodies.

**Figure 2:**
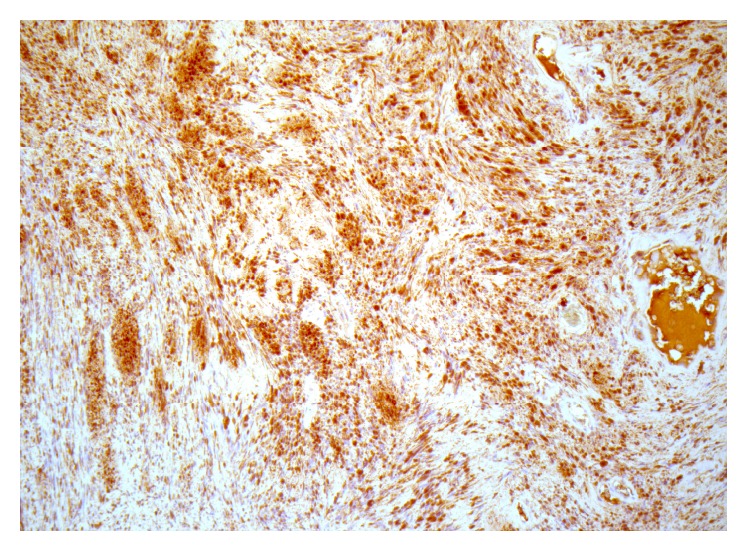
*High power slide of schwannoma*: S100 (immunoperoxidase) staining ×200. High-power view of the lesion demonstrating the characteristic diffuse positive staining with S100 protein.

The diagnosis was made following histological examination of the specimen which demonstrated cytologically bland spindle cells in hypercellular Antoni A and hypocellular Antoni B patterns (Figure 1). Scattered dilated and focally thrombosed blood vessels were visualised which are a feature of these lesions. Staining for S100 immunoperoxidase was strongly positive which is characteristic of schwannoma (Figure 2).

## 4. Discussion

Schwannomas are benign nerve sheath tumours arising from Schwann cells. They comprise 1% of all benign tumours [2]. The lip is the least likely oral location with only twenty documented cases. Despite their rarity, schwannomas should be considered in the differential diagnosis of any lip lump owing to the lip's neural innervation.

In terms of preoperative diagnosis and estimating margins, ultrasound, CT, and MRI can be used but are not considered routine or necessary [3]. In our case no such imaging was performed. Diagnosis is typically made following adequate biopsy. The histological section demonstrated a circumscribed nodule consisting of spindle cells mixed with vascular spaces containing red blood cells and fibrin (Figure 1). No cytological atypia or mitotic activity was noted. Immunohistochemistry for S100 was strongly positive (Figure 2). Findings were consistent with that of a small benign schwannoma. The nodule appeared to be circumscribed and intact suggesting that complete excision had been achieved.

Despite the reassuring histology, lip schwannoma can be histologically misleading and can resemble malignancy [4–6]. However, only three cases of recurrent malignant lip schwannoma have been documented [7–9].

The current consensus is for surgical excision of a conservative nature with no need for margins with the aim being to preserve the nerve of origin [4]. Complete excision should not lead to recurrence. If recurrence does occur one needs to consider whether complete enucleation was achieved or if malignant transformation is a possibility.
