# Corrigendum to “A Lip Lump: An Unexpected Histological Diagnosis of a Lip Schwannoma”

**DOI:** 10.1155/2019/3852428

**Published:** 2019-07-21

**Authors:** Thomas Haigh, Raad John Glore, David Gouldesbrough, Winson Wong

**Affiliations:** ^1^Bradford Teaching Hospitals NHS Foundation Trust, Bradford BD9 6RJ, UK; ^2^Bradford Teaching Hospitals NHS Foundation Trust, University of Leeds, Leeds, UK

In the article titled “A Lip Lump: An Unexpected Histological Diagnosis of a Lip Schwannoma” [1], the name of the second author was given incorrectly as John Raad Glore. The author's name should have been written as Raad John Glore. The revised authors' list is shown above.
